# Clinically important improvement in the WOMAC and predictor factors for response to non-specific non-steroidal anti-inflammatory drugs in osteoarthritic patients: a prospective study

**DOI:** 10.1186/1756-0500-5-58

**Published:** 2012-01-23

**Authors:** Ihsane Hmamouchi, Fadoua Allali, Latifa Tahiri, Hamza Khazzani, Leila El Mansouri, Sanae Ali Ou Alla, Redouane Abouqal, Najia Hajjaj-Hassouni

**Affiliations:** 1 Laboratory of Information and Research on Bone Diseases (LIRPOS), Faculty of Medicine and Pharmacy, Rabat, Morocco; 2 Laboratory of Biostatistical, Clinical and Epidemiological Research (LBRCE), Faculty of Medicine and Pharmacy, Rabat, Morocco

## Abstract

**Background:**

The aims of the present study were first to detect MCID for WOMAC in a Moroccan population, and second, to identify the best pre-treatment predictors on the change of health after treatment by non-specific, non-steroidal anti-inflammatory drugs (NSAIDs), and to evaluate whether the predictors were dependent on the choice of the response criterion.

**Methods:**

The study involved 173 patients with osteoarthritis in whom primary care physicians decided to start treatment with non-selective NSAIDs. Assessments at admission and after 6 weeks were conducted. In order to determine the threshold levels associated with a definition of clinically important improvement, the receiver operating characteristic method was used. Three different measures of response to a 6-week NSAIDs treatment were used: one indirect measure (MCID in the total WOMAC score), one direct measure (transition question) and a combination of both criteria.

**Results:**

Eighty patients (46.3%) reported "a slightly better" general health status compared to that of 6 weeks before NSAIDs treatment. The MCID proportion is a 16.0% reduction in WOMAC. The most stable pre-treatment predictors on the improvement of health after treatment by NSAIDs were the absence of previous knee injury and a high level of education.

**Conclusions:**

In our data, a 16.0% reduction of the total WOMAC score from baseline was associated with the highest degree of improvement on the transition scale category. This cut-off point had good accuracy, and should be appropriate for use in the interpretation of clinical studies results, as well as in clinical care.

## Background

Osteoarthritis (OA) is one of the most common disabilities from which the elderly population suffers, and is projected to be the fourth leading cause of disability worldwide by the year 2020 [1]. A disability may be characterized as the impaired performance of expected socially defined life tasks, in a typical socio-cultural and physical environment [2,3].

A comprehensive assessment of the patient's health status is gaining in importance, now that health care is becoming increasingly evidence-based. As the growing number of the elderly in industrial nations exerts additional pressure on the fiscal resources of health care systems, medical action within strict guidelines is in greater demand [4-6]. One of the key issues for evidence-based and cost-effective medicine is the detection and proof of the effects of a particular intervention. In fact, the ability of an instrument to detect such a small difference is essential in order to quantify the minimal difference that patients and their physicians consider clinically important. The minimal clinically important difference (MCID) can be defined generally as the smallest difference in score that patients perceive as beneficial and which would then mandate, in the absence of troublesome side effects and excessive costs, a change in the patient's management [7]. In particular, when a therapy is ameliorative rather than curative, clinicians need to know whether a small degree of symptom relief is important or trivial from the patient's perspective [8]. For the assessment of interventions in OA of the lower extremities, the Western Ontario and McMaster Universities Osteoarthritis Index (WOMAC) is generally recommended as the most sensitive, condition-specific instrument [9-14].

Symptomatic treatments of OA consist of non-pharmacological as well as pharmacological interventions, including the use of non-steroidal anti-inflammatory drugs (NSAIDs). A major goal of OA treatment is, therefore, pain management to optimize algo-functional features, and to improve the patient's quality of life. NSAIDs reduce inflammation, alleviate pain, and maintain functional activity. Therefore, knowledge of predictors and the identification of patients for whom the probability of treatment success is high at the time of assessment might facilitate the optimization of individual programs. We are interested in identifying baseline risk factors to help clinicians better identify which of their patients evaluated for the first time are likely to make future improvements.

The minimal clinically important difference for WOMAC has not been studied in the Moroccan population. Thus, the aims of the present study were first, to detect MCID for WOMAC in a Moroccan population, and second, to identify the best pre-treatment predictors on the change of health after treatment by non-specific, non-steroidal anti-inflammatory drugs (NSAIDs), and to evaluate whether the predictors were dependent on the choice of the response criterion.

## Methods

### Study design

The ethics committee of Al Ayachi University Hospital approved the study protocol and all patients gave informed written consent prior to their inclusion in the study. This is an ancillary protocol to a prospective non-randomized study that involved 173 patients with OA in whom primary care physicians decided to start treatment with non-selective NSAIDs. Data were collected between January and May 2009 at Al Ayachi University Hospital. The study was specifically designed with inclusion and exclusion criteria that would yield a study population representative of community-based osteoarthritis patients. Eligible patients were aged 18 years or older; had osteoarthritis of the knee (meeting American College of Rheumatology classification criteria); had experienced at least moderate pain in the worst-affected knee (a score of 30 mm or more on a visual analogy scale (VAS) as assessed by the patient) that was judged by the investigator to require treatment with an anti-inflammatory agent to control arthritis symptoms; and had a Functional Capacity Classification of ranging from I to III [15]. Patients were excluded from the study if they had an active gastrointestinal disease, a history of gastric or duodenal ulcer, gastrointestinal bleeding or ulcer perforation, cancer, serious hepatic or renal diseases or any condition precluding NSAID therapy, previous exposure to investigational coxibs and NSAIDs during the past 3 months, and concomitant use of corticosteroids, anticoagulants, or low dose aspirin. Additional criteria for exclusion were intra-articular corticosteroid or intra-articular hyaluronic acid joint injection within 8 weeks before randomization, a known allergy of indomethacin or diclofenac and history of abuse use of alcohol or drug use within 1 year before screening. Pregnant or breast-feeding women were also not eligible. Patients meeting entry criteria received either indomethacin (25 mg) 150 mg daily or diclofenac (50 mg) 150 mg daily for 6 weeks. A clinical evaluation was performed by the investigators at the screening visit, and then again at 6 weeks.

### Data collection and measurements

**Figure 1:**
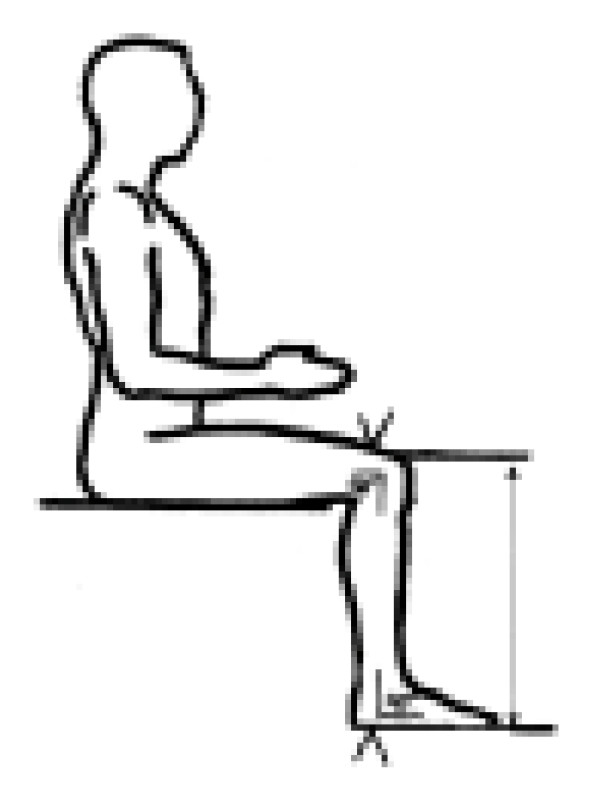
**Body position for the measurement of knee height (16)**. To measure knee height, the knee was bent to a 90° angle, and the distance from the undersurface of the heel (the heel rested on the caliper blade, and sandbags placed under the foot ensured that the foot remained level with the heel) along the calf to the anterior surface of the thigh over the femoral condyles (just proximal to the kneecap) was measured.

At baseline, we collected data related to socio demographic parameters such as age, the number of pregnancies, the level of education, the existence of previous knee injuries and the duration of disease. We asked patients if they have a back pain (Yes/No), currently smoking (Yes/No), and comorbidity (binary): presence of at least 1 comorbid factor: ischemic heart disease, hypertension, diabetes mellitus, renal disease (proteinuria or haematuria) or current cancer. The body mass index (BMI) was calculated as body weight (kg)/height (m^2^). Knee height was measured on the right leg, using a sliding broad-blade caliper, with the subject in the seated position (see Figure 1) [16].

### Western ontario and McMaster universities OA index (WOMAC)

At baseline and after 6 weeks, patients were asked to complete the Western Ontario and McMaster Universities OA Index (WOMAC). The WOMAC Osteoarthritis Index is a disease-specific self-report questionnaire for measurement of the symptoms of OA of the hips and knees. It is reliable, valid, and sensitive to the changes in the health status of patients with knee OA [13]. We used the 3.1 Likert version with five response levels for each item, representing different degrees of intensity (none, mild, moderate, severe, or extreme) that were scored from 0 to 4. The final score for the WOMAC was determined by adding the aggregate scores for pain, stiffness, and function. Scores range from 0 to 96 for the total WOMAC where 0 represents the best health status and 96 the worst possible status. The higher the score, the poorer the function. Therefore, an improvement was achieved by reducing the overall score. The WOMAC has been translated and validated in Arabic [17].

### Transition scale

At the 6-week follow-up, patients had to compare their general health status with that of 6 weeks earlier by the transition questionnaire [18,19]. As described by the authors, the question was "Please imagine how you would have described your health status six weeks ago. How do you feel in general today as compared to six weeks earlier as far as your osteoarthritis is concerned?." The possible replies were "much better," "slightly better," "no change," "slightly worse," or "much worse". This question was used as an anchor to establish the MCID for patients receiving a NSAIDs treatment. We used the answer "slightly better" to establish the MCID for improvement.

### The EuroQol

At baseline, patients were asked to complete the Medical Euroqol-5D (EQ-5D). It is a self-report questionnaire that has two sections: The first part (EQ-5D) consists of five questions covering the dimensions of mobility, self-care, usual activities, pain/discomfort and anxiety/depression, each with three levels of response. The responses to the five items of the EQ-5D can be scored using a utility-weighted algorithm [20] to create a single index of quality of life ranging from -0.59 to 1, which has been recommended for use in economic evaluation. The second part (EQ-VAS) of the EuroQol consists of a 20 cm vertical visual analogue scale (VAS) ranging from 100 (best imaginable health state) to 0 (worst imaginable health state). The EuroQol has been translated and validated in Arabic [21].

### Radiography

Plain radiographs while standing on both legs and the knee extended were taken with a horizontal X-ray beam, using a Fuji FCR capsula XL on a 20 × 25 cm Fuji ST-VI Computed Radiography (CR) imaging plate (Fuji Medical Systems, Tokyo, Japan). Rotation of the foot was adjusted to keep the second metatarsal bone parallel to the X-ray beam. Images were downloaded into Digital Imaging and Communication in Medicine (DICOM) format files with a spatial resolution of 1584 × 2016 pixels (giving a pixel size of 0.01 mm) and 1024 gray levels. Radiographs were evaluated for the presence of OA defined by the Kellgren-Lawrence (KL) scale depicted in the Atlas of Standard Radiographs of Arthritis (0 = normal, 1 = doubtful OA, 2 = minimal OA, 3 = moderate OA, and 4 = severe OA) [22]. This scale is based on the degree of osteophyte formation, joint space narrowing, sclerosis, and joint deformity. The joint space width (JSW) was measured on both the medial and lateral aspect of each knee radiograph with electronic calipers. The minimum vertical distance of JSW was chosen for analysis. To avoid inter-observer variability, the same examiner who was unaware of subject characteristics performed all measurements.

### Statistical methods

Calculation of the required sample size was based on the assumption that indomethacin would reduce the incidence of upper abdominal pain from 40 to 25%, compared with diclofenac with a two-sided test, an alpha level of 0.05, and a power of 80%. The statistical analysis was performed in three steps. First, descriptive statistics were calculated for baseline characteristics. There were expressed as mean (standard deviation) or medians (quartiles) for continuous variables and as percentage distributions for discrete variables. Normality of the data was tested with a one-sample Kolmogorov Smirnov test to indicate the appropriateness of parametric testing. In the second step, the difference between the mean effects measured by WOMAC of the "slightly better" group and the "no change" group was defined as the MCID of improvement. This method has been proposed and applied in different settings [18,23-25]

In order to determine the threshold levels associated with our "a priori" definition of clinically important improvement of WOMAC, the receiver operating characteristic (ROC) method, was used. Transition scale was utilized as an external criterion to distinguish between improved and non-improved patients. This method has the advantage of synthesizing information on the sensitivity and specificity for detecting improvement by an external criterion [26,27]. The Area Under the ROC curve (AUC) in this setting can be interpreted as the probability of correctly identifying the improved patients from non-improved. The area ranges from 0.5 (no accuracy in distinguishing improved from non-improved) to 1.0 (perfect accuracy) [26,27]. According to Swets et al. [28], areas from 0.50 to about 0.70 represent poor accuracy, those from 0.70 and 0.90 are useful for some purposes, and higher values represent high accuracy. From the ROC curves we compute the optimal cut-off point, corresponding with the maximum sum of sensitivity and specificity. The mean effects measured by WOMAC of the "slightly better" group and the "no change" group was defined as the MCID of improvement on the WOMAC global score [6,7,9,10].

The total WOMAC score at the 6-week follow-up minus the score at baseline examination prior to the treatment defined the effect measured by WOMAC. The transition scale assessed the self-perceived change at the 6-week follow up compared to baseline. To determine MCID, the WOMAC effects were related to the transition replies. From the ROC curves we computed the optimal cut-off point, using Youden's index. We estimated the MCID proportion (%), which is the proportion of the sample with a change score exceeding the MCID.

In the last step, three logistic regression models using three definitions of the dependent variable responder were developed. The first definition of response was the MCID improvement (%) on the WOMAC global score. The second definition of responder used the transition scale. Patients who reported a slightly or a much better health status on the transition scale were classified as responders. The third definition of responder required that responders showed an MCID in improvement on the WOMAC global score and reported a health improvement on the transition scale. Comparisons in the change between three categories are carried out using analyses of covariance (ANOVA) and we used Bonferroni adjustment for each two samples categories (womac and transition scale), (womac and both transition scale and womac), (transition scale and both transition scale and womac). Factors found to be significant to the *P *< 0.25 level in univariate analysis because variables close to significance in univariate analysis can become significant in multivariate analysis, and variables that were statistically significant predictors in one of the other models, were included to the model and stayed in the model. To examine whether disease severity is a predictor for response the WOMAC global baseline score was included in the analysis with the dependent variable "responder on the transition scale". It was not included in the analysis with the dependent variable "MCID improvement (%) on the WOMAC global score", because this response definition was derived from the relative change that adjusts for the expected high correlation between absolute change and initial scores. Likewise, the WOMAC baseline score was not included in the analysis with the dependent variable "MCID improvement (%) on the WOMAC global score and responder on the transition scale". Univariate chi-square tests were used to analyze the associations between response and binary independent variables. Discrimination was assessed using the area under the receiver operator characteristic curve (AUC) and calibration was assessed using the Hosmer-Lemeshow goodness-of-fit test for each model using the definitions of responder. This article does not show the results about thresholds

All statistical analyses were performed using SPSS 13.0 for Windows. A significant *P *value of ≤ 0.05 was designated for all assessments.

## Results

### Socio-demographic and clinical characteristics of patients (Table 1)

**Table 1:** Characteristics of study participants

Number	173	
	*Mean (SD)*	*KS Z*
Age (years)	57.1 (10.1)	*0.874*
Weight (kg)	76.2 (12.1)	*0.875*
Height (cm)	155.5 (5.4)	*0.966*
Body mass index (kg/m2)	31.1 (4.8)	*0.770*
Joint space width	3.2 (1.2)	*0.910*
EQ 5D index at baseline	0.2 (0.1)	*0.984*
EQ 5D after treatment	0.6 (0.3)	*0.822*
VAS EQ 5D at baseline	40 (18)	*0.604*
VAS EQ 5D after treatment	67 (18)	*0.597*
WOMAC at baseline	66 (20)	*0.595*
WOMAC after treatment	34 (17)	*0.628*
	*Median (quartiles)*	
WOMAC raw change within transition scale at endpoint		
Much better	13 (12-22)	5.574*
Slightly better	28 (13-39)	3.765*
No change	17 (8-22)	5.318*
Slightly worse	1 (-8-11)	6.450*
Number of pregnancies	4(1-6)	1.366*
Duration of OA (month)	4 (2-10)	2.001*
	*N(%)*	
Female	125 (72.3)	*-*
Existence of previous knee injuries	26 (15.1)	*-*
Existence of back pain	58 (33.5)	*-*
Co morbidities	101 (58.4)	*-*
Smokers	12 (6.9)	*-*
High level of education	61 (35.3)	*-*

*KS *: Komorgorov Smirnov * *P *< 0.05

Normality of the continuous data was tested with the Komorgorov Smirnov test

A total of 221 patients were screened and 173 were included. The most common reason for non-inclusion in the study was an inability to satisfy the clinical inclusion criteria (n = 43). Five patients refused participation. All patients completed the 6-weeks treatment period. The baseline characteristics for 173 patients are shown in Table 1. The mean (SD) age was 57.1 (10.1) years and the mean (SD) BMI was 31 (4.8) kg/m2. The majority was female (72.3%). Of the 173 patients enrolled, 26 (15.1%) reported previous knee injury and 61 (31.7%) had no formal education.

### MCID improvement of total WOMAC score

**Figure 2:**
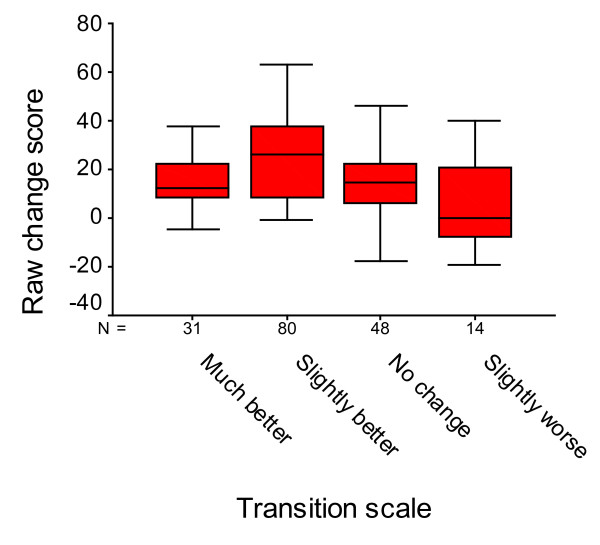
**Box plot of the WOMAC raw change (6-week follow up vs. baseline) within transition scale at endpoint**. Central line, median; boxes, 25th to 75th percentiles; whiskers, 95% confidence intervals.

**Figure 3:**
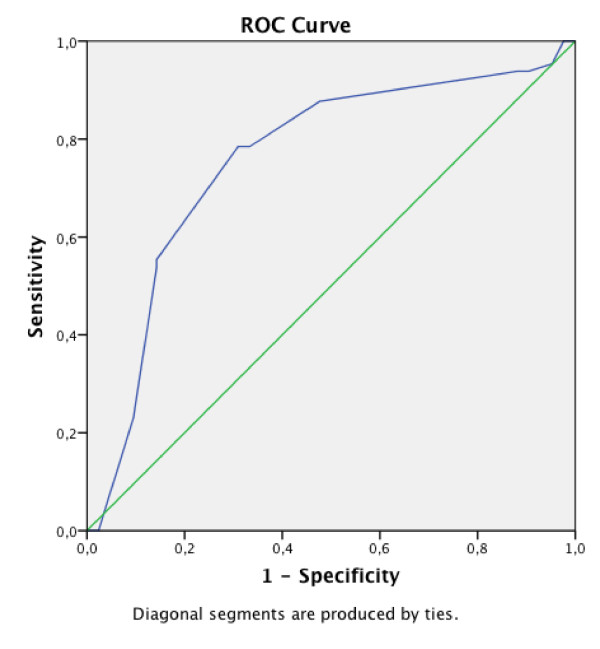
**ROC curve of the WOMAC total score**. A percent change of -16% generated from the ROC analyses were optimal cut-off point associated with our definition of MCID, namely the transition category of "slightly better". The sensitivity and specificity of the cut-off point were 75% and 68% respectively.

The mean changes in total WOMAC score (6-week follow-up versus baseline) stratified by the transition scale, are illustrated by bar charts in Figure 2. More positive scores indicate greater improvement for WOMAC. Eighty patients (46.3%) reported "a slightly better" general health status with that of 6 weeks before NSAIDs treatment and 31 (17.9%) answer that they have "much better" improvement in quality of life. The group of those who answered that they were slightly worse was small (8.1%). The comparison of the change in total WOMAC score among the different groups showed significant difference (one-way analysis of variance, *P *< 0.001; bonferonni (difference between "slightly better" group and "equal, worse" group)). The MCID proportion is a 16.0% reduction in WOMAC. A raw change of -15.5 (AUC 0.881 ± 0.011) and percent change of -16% (AUC 0.889 ± 0.008) genarated from the ROC analyses were optimal cut-off point associated with our definition of MCID, namely the transition category of "slightly better". The sensitivity and specificity of the cut-off point were 75% and 68%, respectively (Figure 3).

### Responder definition

#### Univariate analysis

At baseline, a joint space width > 3.5 mm, a BMI > 31 kg/m^2^, knee height > 49 cm, existence of previous knee injuries, a high level of education and a number of pregnancies > 4 were associated with higher improvement in WOMAC after 6 weeks.

Patients with the lowest level of the total WOMAC score at baseline and the lowest KL Grade had the greatest improvement in transition scale.

Patients with the lowest level of quality of life at baseline (EQ-5D index and VAS) had the greatest improvement in WOMAC and in the transition scale (Table 2).

**Table 2:** Predictors of response to NSAIDs on the univariate level with three different definitions of responder

Independent variable	16% improvement in the WOMAC		Transition		Both criteria	
	**OR**	**IC 95%**	**OR**	**IC 95%**	**OR**	**IC 95%**

Total WOMAC score at baseline	-		0.43*	0.22-0.82	-	
Age > 57 (years)	1.20	0.66-2.16	0.41	0.21-0.77	1.14	0.61-2.13
BMI > 31 (kg/m2)	1.79*	1.02-3.28	0.81	0.43-1.49	1.18	0.63-2.18
Joint space width > 3.5 mm	0.48*	0.23-0.98	1.59+	0.76-3.21	0.61+	0.28-1.30
Knee height > 49 cm	2.46*	1.18-5.13	0.75	0.36-1.51	1.05	0.49-2.25
KL Grade > 2	1.34	0.71-2.54	0.47*	0.23-0.97	1.26	0.68-2.45
Duration of OA > 4 (month)	1.14	0.51-2.56	0.75	0.39-1.72	1.38	0.59-3.21
Previous knee injuries	5.27*	1.83-15.38	2.25+	0.86-5.85	3.92*	1.59-9.67
Co morbidities	0.71	0.38-1-28	0.67+	0.35-1.21	0.60+	0.32-1.13
Existence of back pain	1.77	0.15-19.9	18.2	0.51-24.5	3.39	0.31-38.2
High level of education	2.19*	1.15-4.19	0.63	0.33-1.21	1.72+	0.91-3.25
Number of pregnancies > 4	2.48*	1.06-5.73	0.51+	0.21-1.21	1.92+	0.81-4.51
EQ index at baseline > 0.36	0.5+	0.24-1	1.47	0.73-3.07	0.58+	0.27-1.26
VAS EQ 5D at baseline > 40	0.21*	0.09-0.41	1.56+	0.73-3.32	0.29*	0.12-0.71

+*P *< 0.25 * *P *< 0.05

Continuous variables were dichotomized by median as cut-off

Univariate analysis for the association between "Total WOMAC score at baseline" and the response definitions "16% improvement in the WOMAC" and "both criteria," respectively, are not represented because these response definitions adjust for the expected high correlation of WOMAC baseline score and the change of the WOMAC

#### Multivariate analysis

1. Responder definition "16% (MCID) improvement in the WOMAC." Statistically significant predictors for a better outcome were high level of education (no formal education/higher than no formal education) (OR = 3.77; IC: 1.12-12.7) and absence of previous knee injury (OR = 4.55; IC: 1.46-14.8)

2. Responder definition "improvement on the transition scale" (data not shown). In this category no factor was a statistically significant predictor for improvement

3. Responder definition "16% improvement in the WOMAC and improvement on the transition scale." Statistically significant predictors for a better outcome were absence of previous knee injury (OR = 10.27; IC: 2.08-50.6), low knee height (OR = 3.34; IC: 1.05-10.5) and high level of education (OR = 3.7; IC: 1.01-13.41)

#### Comparison of prediction models with different responder definitions

The model with the responder definition that required responders to have "a 16% improvement in the WOMAC and improvement on the transition scale" had the highest value for the discrimination (AUC = 0.84). This model had also the best calibration of fit (PHL = 0.53) (Table 3). The model with the restrictive responder definition that required responders to have an improvement on the transitions scale had the lowest value for the discrimination and calibration (AUC = 0.68, PHM = 0.25) data not shown.

**Table 3:** Comparison of predictors for OA treatment by NSAIDS with different definitions of responder

	N (%)	AUC	P (HL)	Predictors	OR	95% CI	*P*	R2
16% improvement*	92 (53.2)	0.77	0.51	High level of education	3.77	1.12-12.7	0.03	0.34
				No Previous knee injury	4.55	1.46-14.8	0.001	
Both criteria+	65 (37.6)	0.84	0.53	No Previous knee injury	10.27	2.08-50.6	0.004	0.47
				Knee height	3.34	1.05-10.5	0.040	
				High level of education	3.7	1.01-13.41	0.047	

*Patients with an MCID on the total WOMAC score (16% change)

+Patients who answered "slightly better" or "much better" to the health transition question and have an MCID on the total WOMAC score (16% change)

After adjusting of Age > 57 (years), Body mass index > 31 (kg/m2), Knee height > 49 cm; Joint space width > 3.5 mm; Existence of previous knee injuries; Co morbidities; high level of education (no formal education vs. higher than no formal education); Number of pregnancies > 4; EQ 5D index at baseline > 0.36; VAS EQ 5D at baseline > 40

## Discussion

In our study, a 16.0% reduction of the total WOMAC score from baseline was associated with the "slightly better" degree of improvement on the transition scale category, and this cut-off point had good accuracy.

The concept of the minimal clinically important difference (MCID) has been proposed to refer to the smallest difference in a score that is considered to be meaningful or clinically important. In our study, the receiver operating characteristic (ROC) method was used. This method has the advantage of synthesizing information on the sensitivity and specificity for detecting improvement by an external criterion.

In fact, the greatest difficulty in assessing the threshold of symptomatic clinical importance is identifying an appropriate reference standard [7] have asked individuals to introspect on life experiences and directly calibrate health status measures. Redelmeier et al. [8] took the patients' perspective and asked patients to evaluate their health relative to others. In this study, we have assessed thresholds by correlating scores on a health status index to an external question: "transition scale."

Several studies have shown that patients tended to rate themselves as less disabled than each other [7,8]. In our study, we found that the threshold of symptomatic difference was smaller if patients were less disabled. This optimistic bias in subjective comparison ratings resembles several other cognitive phenomena. People can adapt to their disabilities and become reluctant to exchange their current problems for an alternative situation [8,29]. Together, these cognitive phenomena highlight pitfalls of assessing a patient's quality of life, both in research as well as in the doctor-patient relationship.

In our study, we estimated the MCID proportion (%) whitch is the proportion of the sample with change scores exceeding the MCID by the ROC analysis. In our study, we found the same result of MCID using ROC curve by correlating WOMAC scores to an external question: "transition scale" that when calculating the difference between the before-after treatment of the WOMAC total score (28 (change score of the slightly better group)--17 (change score of the unchanged group) = 11 score points. 11/66 (66 = baseline WOMAC score) = 16.7%). This is a coincidence that we cannot explain.

The magnitude of the improvement seen in WOMAC in our study after 6 weeks (16%) reflects a slightly poorer condition than the one reported following a similar methodology. Indeed, previous studies have shown an 18.0% reduction in WOMAC as the percentage of patients with a MCID [30,31]. In our study, X-ray examinations were performed at baseline, and showed that 67.7% of patients presented higher than KL grade 3. Furthermore, owing to the fact that above 30 mm on the VAS of pain was an inclusion criterion of the study, the severity of OA in our cohort could be considered as moderate to severe.

The best response definition in this study was a combination of the transition question and percentage change in WOMAC total score. This model had a better ability to discriminate between responders and non-responders than the other two models. In fact, to ask the patient about a health transition is simple and intuitive, and patients with a very good or a very bad health status can deteriorate or improve, respectively. But a single transition question cannot be considered as a stable measure. Taking into account the high mean age of our samples and their low level of education, this option would have made answering the questionnaire far more complicated. Further studies has shown that the MCID proportion is a reliable and valid measure, and may be more stable than a single transition question because of absence of optimistic bias [18,19]. Therefore, patients with baseline scores far above the average (bad health) show higher improvements in the change score compared to patients with a good health status [18,31]. This is a well-known phenomenon: a sick patient has higher potential to improve and the regression-to-the-mean effect. The more the patient's condition is serious, the more the improvement will be important because the difference between the before-after treatment will be considerable.

In our report, the most stable pre-treatment predictors on the improvement of health after treatment by NSAIDs in Moroccan osteoarthritis patients were the absence of previous knee injury and a high level of education, because they were confirmed across two logistic regression models with different definitions of response as the dependent variable.

A better outcome for highly educated patients was found in studies that evaluated the outcome of joint arthroplasty [31,32]. On the other hand, joint injury is a well-known risk factor for knee OA and is associated with increased severity of osteoarthritis [33-36]. This is in line with our results. However, given that high level of education and previous knee injury are historic events, this raises the issue as to whether these are modifiable factors. Since this is obviously not the case, it seems interesting to emphasize the therapeutic patient education especially in patients with low level of education.

The finding that presence of previous knee injuries is associated with higher improvement in WOMAC total in the univariate analysis but absence of previous knee injuries is associated with higher improvement in WOMAC total in the multivariate analysis may be confusing. It is probably due to the existence of confounding factors. When a variable is related to another variable of the model, it may be obscured in univariate analysis (not significant) and prove to be in multivariate analysis. In our models, it may be the case of "previous knee injury" and "joint pace width."

Our study has some limitations. The patients in the study were subjects for whom primary care physicians identified the need to start treatment with NSAIDs. This fact suggests strongly that these patients were symptomatic at the time of the quality of life assessment. Because OA may be characterized by phases of flares and respite, the results we observed might be slightly overestimated compared with the whole population of patients with OA. A possible limitation was that we employed one transitional question, not one for each domain, as it has been suggested [37]. This would have increased the length of the questionnaire and increased the rate of missing patients or items. Another question that could be asked about our results is the absence of a direct comparison with a control group. However, a large set of complete data on socio-demographic variables was entered into the regression models, and in addition, clinical measurements were taken.

## Conclusions

In our data, a 16.0% reduction of the total WOMAC score from baseline was associated with slightly better improvement on the transition scale. This cut-off point had good accuracy, and should be appropriate for use in the interpretation of clinical studies results, as well as in clinical care. The observation that patients with a high level of education and an absence of previous knee injury are the best responders to NSAIDs in Moroccan osteoarthritis may be interesting to adopt and individualize the treatment of patients who are, at present, less likely to respond and to emphasize the therapeutic patient education especially in patients with a low level of education.

## Abbreviations

NSAIDs: Non-steroidal anti-inflammatory drugs; OA: Osteoarthritis; MCID: Minimal clinically important difference; WOMAC: Western Ontario and McMaster Universities Osteoarthritis Index; VAS: Visual analogy scale; BMI: Body mass index; EQ-5D: Medical Euroqol-5D; KL: Kellgren-Lawrence; JSW: Joint space width; KS: Kolmogorov-Smirnov; AUC: Area Under the ROC curve; H-L: Hosmer-Lemeshow

## Competing interests

The authors declare that they have no competing interests.

## Authors' contributions

All authors participated at the study as following: FA, NHH conceived the study and supervised its design, execution, and analysis and participated in the drafting and critical review of the manuscript. IH, FA and RA did data management and statistical analyses. All other authors' enrolled patients, participated in data acquisition and critical revision of the manuscript. IH wrote the paper with input from all investigators. All authors declare that we have seen and approved the final version of manuscript.
